# Assessment of the Welfare of Working Horses: Combining Clinical Evaluations with Indirect Indicators

**DOI:** 10.3390/vetsci13030274

**Published:** 2026-03-16

**Authors:** Abdallah A. Basher, Abdelkareem A. Ahmed, Davies M. Pfukenyi, Hao-Yu Liu, Mohamed Osman Abdalrahem Essa, Hosameldeen Mohamed Husien, Ahmed A. Saleh, Saber Y. Adam, Demin Cai

**Affiliations:** 1 College of Animal Science and Technology, Yangzhou University, Yangzhou 225009, China; hammodavet@gmail.com (A.A.B.); 007725@yzu.edu.cn (H.-Y.L.); 008643@yzu.edu.cn (H.M.H.); 2 Biomedical Research Institute, Darfur University College, Nyala 155, Sudan; kareemo151@gmail.com; 3 Department of Veterinary Science, Faculty of Animal and Veterinary Science, Botswana University of Agriculture and Natural Resources, Gaborone Private Bag 0027, Botswana; dpfukenyi@buan.ac.bw; 4 College of Veterinary Medicine, Albutana University, Rufaa 22217, Sudan; mohosman0999@gmail.com; 5 Animal and Fish Production Department, Faculty of Agriculture (Al-Shatby), Alexandria University, Alexandria 11865, Egypt; elemlak1339@gmail.com

**Keywords:** animal welfare, animal behavior, South Darfur, Sudan

## Abstract

Assessing the welfare of the working horses is crucial for identifying health and management issues and implementing effective solutions. This study evaluated the welfare of working horses in South Darfur, Sudan, by integrating clinical examination with indirect indicators through owner interviews. A total of 400 horse–owner pairs were included. Most horses (61.7%) worked every day, and 77% of owners reported using whips to encourage movement. Clinical assessment revealed that 29.0% of horses were thin, over half had dirty coats, 50.5% harbored external parasites, 43% had abnormal discharges, 25.0% showed poor hoof health, and many exhibited gait abnormalities. Behavioral assessments indicated that fewer than 40% of horses displayed alert attitudes, while over half responded indifferently to stimuli. Horses working daily tended to be thin, have skin lesions, and show gait problems, reflecting poor welfare. Horses with better attitude responses were more frequently fed and worked fewer days per week. Overall, the findings highlight poor physical, behavioral, and management conditions among working horses, emphasizing the urgent need for owner education, training programs, and further research to improve their welfare in the study area.

## Simple Summary

This study used clinical tests and owner interviews to evaluate the welfare of working horses in South Darfur, Sudan. According to the findings, most of the horses worked every day. We found that 77% of owners used beating to encourage horses’ activity, while 45% of owners never consulted veterinarians. According to clinical data, 43% of horses had abnormal discharges, 29% were thin, and more than half had dirty coats and external parasites. In addition, many had gait problems, and 25% had poor hoof health. In terms of behavior, more than half reacted indifferently to stimuli, and fewer than 40% displayed alertness. Horses that worked every day were more likely to be thin, with skin lesions and gait issues, indicating poor welfare. Horses with better attitude responses ate more frequently and worked fewer days per week. Overall, the horses demonstrated poor physical and management characteristics, underlining the need for improved owner awareness, practical education, training programs, and additional research to improve their welfare in the region.

## 1. Introduction

Working animals such as horses, donkeys, and mules are vital to human livelihood in many parts of the world, particularly in developing countries [1]. Non-governmental organizations (NGOs) assert that working animals, including horses, make a significant contribution to the socioeconomic well-being of their communities; however, there is limited published scientific evidence to support this claim [2]. In many impoverished societies, working animals are responsible for daily activities and tasks that are critical for their owners’ survival, including transportation and healthcare [3] They are commonly used for transportation, labor, sport (jumping, racing), riding, and breeding. According to 2014 data from the Food and Agriculture Organization of the United Nations (FAO), Africa had a population of 26.03 million equids, including 1.02 million mules, 6.06 million horses, and 18.9 million donkeys [4].

In Sudan, both urban and rural communities rely on horses for carrying firewood, transporting goods and water, and other labor-intensive tasks, especially in rural areas [5]. Horses serve as draught and pack animals, assist in agricultural work, and provide riding services. They are often owned by economically disadvantaged individuals with incomes below the international poverty line [6]. Horses are particularly valuable in areas that are inaccessible to motorized vehicles, where alternative transportation methods are costly, even for short distances with light loads [7]. Although some farmers in Darfur keep sturdy animals like camels for labor, meat, and milk, equines remain the primary source of draught power [5].

Despite their critical role, working horses in these regions are often subjected to poor husbandry, which negatively affects their health, emotional status and work capacity [1]. Our previous study of working horses in middle and northern Darfur indicated that these animals are vulnerable to a numerous health problems due to inadequate veterinary care, neglect, and abuse [5]. Husbandry practices have been identified as significant psychological stressors for horses [8]. For ridden horse, additional stress can result from exposure to new stimuli, separation from companion animals, and interaction with unfamiliar horses [9]. Pain from injuries, improper training methods, ill-fitted tack, and imbalanced or heavy riders also compromise welfare [10]. Common harmful practices include poor handling during loading and unloading; working in hot and dusty environments; inadequate shelter, care and management; working in hot and dusty conditions; crude castration; the use of restraining devices; long-distance walking; and overloading [11].

In countries where animal welfare is regulated, horses can live for 20 to 30 years, with some individuals living over 40 years [12]. However, the working lifespan of horses in Darfur is typically limited to 4–6 years due to the lack of veterinary care, abuse, and harsh working conditions [13]. Recognizing the need for standards, the World Organisation for Animal Health (WOAH) recently established the first welfare guidelines for working horses used in traction, transportation, and commercial activities [14]. The concept of “fit and feeling good” emphasizes that animal welfare encompasses both psychological and physical well-being. Emotional welfare aims to minimize mental states such as fear, pain, and distress. Physical welfare is determined by health and is influenced by disease and injury [13].

Despite the critical role of working horses in South Darfur, their husbandry and welfare conditions are poorly documented. This study aims to evaluate the welfare of working horses in South Darfur, Sudan, by integrating direct clinical assessments with indirect indicators, thereby providing a comprehensive understanding of their health and well-being in working environments.

## 2. Materials and Methods

### 2.1. Data Collection

This study was conducted in South Darfur State, located between latitude 8° and 14° north and longitude 22° and 28° east. This region borders South Sudan and Central African Republic to the south, North Darfur to the north, Central Darfur to the west, and East Darfur State to the southwest [11]. The research was carried out from 31 May to 2 October 2024, with the aim of assessing the key welfare challenges, health issues, and other factors affecting working horses in the area. The welfare assessment of horses was conducted using an established welfare evaluation protocol based on previously published guidelines (Table 1, Table 2 and Table 3) [15,16].

### 2.2. Sample Size Determination and Inclusion Criteria

To ensure the representativeness of the sample, working horses and their owners/users were eligible for inclusion in the study if they met the following criteria: (1) The horse was actively used for work purposes (transportation, agriculture, riding, or others) at the time of the study; (2) the owner or user was present and consented to participate; and (3) the horse was available for physical examination on the day of visit. Horses and their owners were selected randomly from various locations within South Darfur State, with breed information being recorded for each horse.

The sample size was determined using the formula for cross-sectional studies, i.e., n = Z^2^PQ/d^2^, where Z = 1.96 for the 95% confidence interval, P = estimated prevalence of poor welfare indicators (50% to obtain maximum sample size), Q = 1 − P, and d = margin of error (5%) [17]. This calculation yielded a minimum required sample of 384 horses. Based on local veterinary authority records, the estimated total population of working horses in South Darfur State exceeds 10,000, thus validating the sample size.

### 2.3. Medical/Clinical Data Collected

The health status, physical condition, and behavior of the working horses were assessed by a well-trained veterinarian following a standardized protocol (Table 1). The horses’ sex was determined by examining the external genitalia, and their ages were classified into three categories: <5, 5–10, or >10 years [18]. Body condition scores (BCSs) were assigned using a traditional 5-point scale, ranking from 1 (very thin) to 5 (very fat). The coat, skin conditions, lesions, and gait abnormalities were recorded as either present or absent. Hoof health was categorized as either adequate or inadequate, based on whether any indicators were altered or remained within normal limits. Skin condition, including any presence of external parasites and the quality of the coat, was evaluated based on established criteria [19].

### 2.4. Measurement the Behaviour Status of the Horses

The behavior of the horses was assessed using a protocol designed for welfare evaluation, as described in earlier studies (Table 2) [16]. The horses’ general attitudes (alert and/or depressed), their reactions to the researcher approaching, their behavior during a walk-by (moving alongside the horse), and their response to chin contact were all evaluated. Additionally, biting and kicking behaviors were observed when the horse was touched by the researcher. A bite attempt was categorized as “yes” if the horse bent its head toward the researcher and attempted to bite, and “no” otherwise. Kicking behaviors, if present, were noted as occurring with either the front or hind legs [5]. Each horse’s response was documented accordingly.

### 2.5. Owner’s Survey/Indirect Indicators

Horse owners were interviewed face-to-face using a structured, standardized questionnaire (Table 3) to gather information on their demographics (sex and age), the working habits of their horses (frequency and type of work), feeding and watering routines, use of tools to encourage movement, shoeing and trimming frequency, and veterinary consultations. Participation was voluntary, and no financial compensation was offered. Owners were informed about the study objective and assured that their responses would be used exclusively for research purposes. In the event of a declined participation, the next willing participant was approached, until the required sample size was achieved [20,21].

**Table 1:** Description of the observation protocol and clinical examination categories.

Indicator	Categories	Explanation
Horse sex	Male/Female	The sex of the horse was determined by examining the external genitalia and classifying as male/female [20].
Age	3–5 years 5–10 years >10 years	Based on the horse’s front teeth, the animal’s age was determined.
Body Condition Score	Very thin Thin/Good Fat/Very fat	A five-point scale, ranging from 1 very thin to 5 very fat, was used to evaluate the horses using the criteria listed earlier [15,22,23,24], and the animals were examined from every angle without being touched. Horses whose ribs, lumbar vertebrae, and pelvic bones were clearly visible from a distance were described as very thin. Horses with easily noticeable bones, although they may have a slight fat covering, who while not as extreme as “very thin” horses still had a clear lack of adequate fat reserves and some visible bony prominences were described as thin. Horses with moderate fat covering and bones that were typically not visibly prominent were described as good. Horses categorized as fat presented a fleshy appearance, with ribs becoming difficult to observe due to a thicker layer of subcutaneous fat. Very fat was described as a visible crease or groove down the horse’s back, with heavy fat deposits being noticeable, particularly at the withers, shoulders, and tailhead, while the ribs were very difficult or impossible to observe due to extensive fat coverage.
Skin wounds and scars	Absent, Neck Chest, Back Hind Quarter, Tail Base	Lesions were noted with relation to anatomical location as part of the examination of wounds and scars if they were present.
Coat condition	Clean Dirty	The horse’s coat was characterized as clean if the hair coat was uniform, appeared healthy (shiny), and was dried of sweat, while a dirty coat was if the horse’s hair was mixed with the hair of other animals (such as on mud or feces) [25].
External parasites	Present Absent	If any species of ectoparasites were discovered on the horses’ hair or skin, the finding was recorded as present, and absent if none found [19].
Hoof health	Adequate Inadequate	Hoof quality, shape and conformation were evaluated. Hoof health was considered adequate if it was smooth and spherical and free of cracks or missing pieces and did not exhibit any flaws in the hoof capsule [25,26].
Gait abnormalities	Present Absent	Evaluated by watching the horse walk in a straight line for roughly 20 m. The researcher made an assessment of any lameness, uneven stride, unwillingness to bear weight on one or more limbs, uneven head-nodding, or hip movement; if any of these occurred, it was documented as present, and if none were observed, it was documented as absent [25].
Discharges from orifices	Nasal/Ocular Mouth/Absent	The external orifices of the horses were assessed for any discharges, and if present, they were recorded for each orifice [5].

**Table 2:** Description of the behavioral status of the working horses in the study area.

Indicator	Categories	Explanation
General attitude	Alert Depressed	The horse was observed from a distance of 3–5 m and viewed from a lateral (side-on) position. The following categories were applied to the horse’s response: It was classified as alert when the horse was focused and reacted to outside stimuli (e.g., wide-open eyes, motion of the head, tail, ears, and/or skin to ward off flies). It was classified as depressed when it showed signs of reduced response to environmental cues, including a drooping head, half-closed eyes, complete or partial cessation of tail and skin motions to fend off insects, and reduced ear movement [16,26].
Approximation test	Indifference Friendliness Avoidance Aggressiveness	The researcher halted at about 30 cm from the horse’s head, approaching at an angle of about 20° to the horse’s body’s sagittal plane. The horse’s reaction was recorded by the researcher as soon as the halting was done. Responses were recorded as follows: Indifference: Depressed or relaxed body posture and facial expression (with or without the ears moving, relaxed lips, sometimes half-closed eyes), without attempts to approach or move away from the researcher. Friendliness: Movement of the head in the direction of the researcher, a calm expression, eyes that were open normally, ears that were turned forward, and no wrinkles around the mouth or nose. Avoidance: The horse was movable and had a tense appearance, with its head held high, eyes wide open, and lips pursed. It may also turn its head or make an attempt to get away from the researcher. Aggressiveness: The horse tried to kick or bite; the eyes were open very wide, and the head was turned towards the researcher; its nostrils were dilated; it may wrinkle its mouth or paw or stomp the ground [16].
Walk-down-the-side	Indifference Friendliness Avoidance Aggressiveness	The researcher went beside the horse, starting from the shoulder and moving toward its back and rear while keeping a 30 cm spacing from its body, and then recorded the horses’s reaction. The horses’ reactions were correctly categorized as in the approximation test [16].
Chin contact	Accept Avoid	The researcher slowly put his/her hand under the horse’s chin to see if it would accept or reject the contact. Horses’ reactions to contact were recorded as either accepting it or avoiding it.
Biting and kicking	Yes No	If the horse turned its head in the direction of the researcher and tried to bite, then the attempt was a yes; otherwise, it was a no. Kicks were carried out with either the front or back legs, or none at all [26].

**Table 3:** Description of the owner/user survey (indirect indicators).

Indicator	Categories	Explanation
Sex	Male/Female	Sex of each participant was noted and recorded.
Age	10–25 26–40 41–55 >55	Each participant was asked to indicate his/her age category, and the age category was recorded.
Daily feeding and watering frequency	Once daily Twice daily >Twice daily	The owner was asked how frequently their horses were fed each day, as previously mentioned [5]. The owner was asked about the daily watering frequency of their horses [20].
Method/tool used to encourage horse movement	Method/tool type	Owners were asked about the method/tool they use to encourage horse movement.
Type of work done by horses	People transportation by cart Goods transportation by cart Riding Agricultural purposes Others	All of the participants were asked about the daily work type of their working horses.
Frequency of shoeing and trimming	Shoeing	The owner was asked how frequently the horse is shoed.
	Between 1 and 2 months	
	>2 months	
	Not done	
	Trimming	The participant was asked about the frequency with which his/her horse was trimmed, as described earlier [5].
	Every 15 days	
	Between 15 and 30 days	
	>30 days	
	Not done	
Person responsible for shoeing and trimming	Farrier Owner Not done	Questions about the primary person in charge of shoeing and trimming the horse were posed to the owner.
Veterinary consultation	<1 year >1 year Never	The owner was asked about the last time a veterinarian inspected the horse. The response was divided into three categories, as described earlier: never (if the horse had never had a vet check), less than a year ago, or more than a year ago [20].

### 2.6. Data Analysis

Data from both direct and indirect welfare indicator data were classified and imported into SPSS (IBM Crop, New York, NY, USA, Version 21). The data analysis focused on generating descriptive statistics (frequencies and percentages) related to the indirect indicators, obtained through horse owner interviews, direct observations and clinical examinations of horses and the behavioral status of the horses. The association between clinical information and indirect indicators was examined using the Chi-squared test and Fisher’s exact test. A statistical level of *p* < 0.05 was considered significant.

## 3. Results

### 3.1. Characterization of the Sample and Results of the Clinical Examination

**Figure 1:**
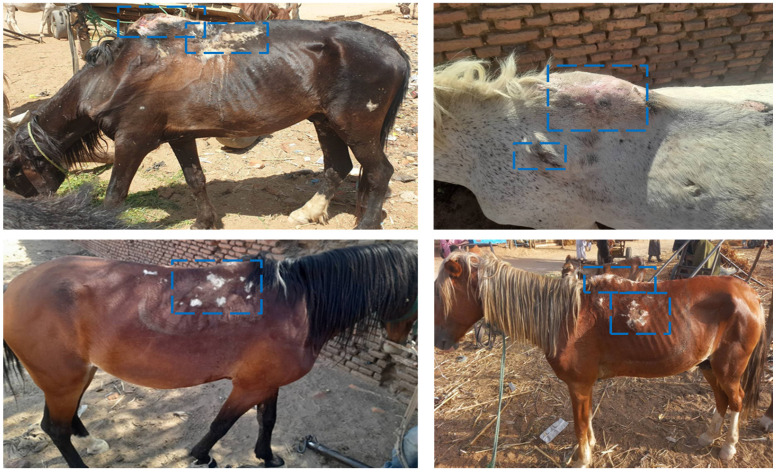
These photos have been taken by Dr. Mudathir and Dr. Saber. Inside the blue boxes show the severity of the wound, scarring, and hair loss on the withers and back region of the working horse due to pressure injuries from improper equipment.

A total of 400 working horses and their owners/users were examined in this study, and all horses were of the indigenous Western Sudan Pony Breed. The majority of horse owners were male (95.8%), with most being aged 25 years or older. Clinical examination (Table 4) revealed that most horses were over 5 years old, and approximately three-quarters of them were stallions. Half of the horses had a good body condition, while one-third were classified as thin or very thin. Regarding health conditions, a substantial proportion of horses exhibited skin wounds on various body parts, with many showing poor coat condition and infestations of external parasites. Approximately 25% of the horses displayed signs of poor hoof health, and nearly 25% exhibited abnormal gait, along with nasal, mouth, and ocular discharges. Figure 1 shows a severe shoulder wound and scar resulting from improper equipment.

### 3.2. Indirect Indicators (Owner Survey/Interview) Results

The results of the interviews with horse owners are summarized in Table 5. The majority of the owners of working horses were male, with most being aged 26 years or older. Most owners reported working with their horses every day of the week, while a smaller potion worked their horses 3–4 days or 1–2 days per week. Approximately one-third of the owners used their horses for transporting people and goods by cart, while about a quarter indicated using them for riding animals.

In terms of care, the majority of owners fed and watered their horses at least twice a day. A significant number of owners reported using whipping as a method to encourage horses. Approximately 45% of the owners reported not consulting a veterinarian, while about 37% sought veterinary advice on an annual basis.

### 3.3. Association Between Clinical Information and Indirect Indicators

The relationship between clinical findings and indirect welfare indicators was assessed. Horses working every day of the week (61.7%) was significantly associated with a thin body condition score (X^2^ = 5.41; *p* = 0.04), the presence of skin wounds (Fisher’s exact test, *p* = 0.001), skin scars (Fisher’s exact test, *p* = 0.02), inadequate hoof health (Fisher’s exact test, *p* = 0.04), and the presence of abnormal gait (Fisher’s exact test, *p* = 0.03).

Additionally, owners who reported never consulting a veterinarian (44.5%) were significantly more likely to have horses with external parasites (X^2^ = 5.72; *p* = 0.01) and orifice discharges (X^2^ = 4.31; *p* = 0.02). The absence of regular shoeing practice (95.5%) was significantly associated with inadequate hoof health (X^2^ = 5.42; *p* = 0.03).

There was no significant association between the presence of gait abnormality (24.5%) and trimming practice (Fisher’s exact test, *p* = 0.08). The condition of the horses’ coat (53.0%) was not significantly associated with the variable of frequency of work (1–2 days per week, Fisher’s exact test, *p* = 0.09; 3–4 days per week, Fisher’s exact test, *p* = 0.12; or daily work, Fisher’s exact test, *p* = 0.07). However, horses having an alert attitude (65.5%) was significantly associated with frequent feeding (more than twice per day, Fisher’s exact test, *p* = 0.001), more frequent watering (more than twice per day, Fisher’s exact test, *p* = 0.02), and working 1–2 days per week (X^2^ = 6.37; *p* = 0.04).

### 3.4. Behavioral Status of the Horses

Less than 40% of the horses exhibited a depressed attitude in response to the researcher. Regarding their reactions to the researcher’s approach, over 50% of horses displayed indifferent responses, while around 27% showed friendly responses. For the walk-down-the-side variable, most horses demonstrated indifference, with a smaller proportion displaying friendliness. Some horses showed avoidance behaviors, and few displayed aggressiveness. Moreover, this study revealed that the majority (>90%) of the working horses did not exhibit any biting or kicking behavior toward the owner and researchers (Table 6).

## 4. Discussion

Working horses play a crucial role in improving the lives of the world’s poorest populations, both directly and indirectly [16]. These animals significantly contribute to economic activities, including the transportation of agricultural products, water, goods, people, and building materials. Additionally, they provide draught power for farming and other labor-intensive tasks [27,28]. Therefore, identifying and addressing the welfare issues that affect working horses should be a priority.

In this study, it was observed that most horse owners/users were males, which aligns with findings from Mamo et al. [29], who reported that 83.8% of horse owners were males. Therefore, the likelihood of men owning working horses is higher than that of women owning work horses. This could be due to culture and religion of the study areas, where is rare for women to work when there are men in the family. Furthermore, our study found that the majority of the owners were between 41 and 55 years. This finding is in disagreement with Popescu et al. [25], who reported that most horse owners in their study were middle-aged (19–45 years), suggesting that age distribution may vary due to regional differences in social and economic factors.

Similar to previous research by Ashinde et al. [30], our study found that the majority of horse owners worked their horses every day of the week. This may reflect the economic and environmental challenges faced by the population in Darfur, where working horses are indispensable for daily tasks [5]. Regarding horse care, most owners in our study reported feeding and watering their horses at least twice a day, indicating a relatively high level of knowledge about basic animal husbandry. These results are consistent with the findings of Adam et al. [5] and Luna et al. [20]. However, a study by Mekuria S. and Abebe R. [13] found that only 24% of owners fed their horses twice daily, which may be due to variations in geographic location, economic status, and the availability of appropriate feed.

Horses’ age is a significant factor influencing their welfare. McLeod [31] has noted that horses perform optimally between the ages of 4 and 12, reaching zootechnical maturity around four years of age. Initiating work before this age increases the risk of earlier mortality and morphological defects like drooping backs [32]. After 12 years of age, a horse’s working efficiency tends to decrease [31]. In our study, more than half of the working horses were older than 10 years, with fewer than 20% between 3 and 5 years old. This suggests that a significant number of horses are being used for labor past their peak working years, while a small proportion are put to work before reaching full maturity [32].

The body condition score (BCS) of horses is a key indicator of their welfare. In this study, we reported that (29%) of horses had a thin or very thin BCS, consistent with the findings of Mekuria S. and Abebe R. [13], who found that 29% of horses were classified as thin during BCS evaluation. A thin body condition can result from various factors, including poor nutrition, excessive work, parasite infestations, and diseases [1]. Notably, the only factor that was significantly associated with a thin BCS was the frequency of work—horses that worked every day of the week were more likely to be classified as thin. This finding aligns with the observation by Adam et al. [5], who reported that 42.7% of horses had dirty coats, suggesting that the condition of the horses in their study was better. Differences in coat conditions may be due to variations in housing systems, grooming practices, or owner awareness. Horses who are housed in cleaner, well-maintained environments are less likely to develop dirty coats, reflecting better overall welfare and management. Horses who are owned by knowledgeable caretakers who follow regular grooming routines tend to have a coat in a better condition.

Hoof health is another critical aspect of working horses’ welfare. In our study, 25% of horses had inadequate hoof care, which can be attributed to a lack of farrier training, limited veterinary services, and insufficient owner knowledge. Previous studies have reported similar issues, with hoof problems and lameness being prevalent in working horses [15]. Variations in grazing and labor practices across regions may explain the discrepancies in the prevalence of hoof abnormalities.

Ectoparasite infestations were found in approximately 50% of the horses in this study, which is notably higher than the 16% reported by Biswas et al. [33]. Several factors may contribute to the higher prevalence observed in our study, including a lack of awareness about healthcare, poor nutrition, and irregular or nonexistent parasite control practices [33]. Inadequate veterinary care may further exacerbate the problem. Indeed, a significant association was found between the presence of ectoparasites and the fact that 44.5% of horse owners never sought veterinary consultations for their horses. This suggests that the lack of professional care and guidance on parasite management is a critical factor in the high prevalence of ectoparasite infestations among the working horses in South Darfur.

Behavioral assessments are valuable for evaluating the welfare of working horses, as they reflect the animal’s interactions and the quality of its relationship with its owner [1,19,^20^]. In our study, more than half of the horses displayed indifferent responses during the approximation and walk-down-the-side tests, which may indicate poor handling and inadequate owner–horse relationships. While many horses exhibited alert and sociable behaviors, the prevalence of indifference could suggest a lack of positive interaction or neglect. Consistent with pervious findings [20], the high prevalence of alertness in our study suggests that many horses are not suffering from extreme distress, but the indifferent responses might indicate underlying issues in the quality of care that they receive.

A limitation of this study is the potential for bias in the response provided by owners during these surveys. Owners may have given answers that they believed the researchers wanted to hear, leading to over-reporting of positive behaviors and care practices. Another limitation is the absence of questions regarding the owner’s emotional attachment to the horses, which may affect their approach to horse care; understanding whether owners view horses as family members or primarily as working tools would provide additional insight into their behavior and welfare.

## 5. Conclusions

This study revealed that various welfare issues, including poor trimming and shoeing practices, lack of veterinary consultations, dirty coats, ectoparasite infestations, and aggressive behaviors, are prevalent among working horses in South Darfur. These findings suggest that the horses in this region suffer from poor husbandry practices. Improving owners’ awareness through education and training programs for both horse owners and local farriers is essential for enhancing the welfare of working horses. Further research and practical interventions are needed to address these welfare challenges and improve the quality of life for working horses in study area.

## Figures and Tables

**Table 4:** Summary of findings on observation protocol and clinical examinations of the horses (n = 400).

Variable	Categories	Number	Percentage (%)
Sex	Male Female	303 97	75.8 24.2
Age	3–5 years 5–10 years >10 years	61 112 227	15.3 28.0 56.7
Body condition score	Very thin Thin Good Fat	15 116 199 70	3.8 29.0 49.8 17.4
Skin wounds	Absent Neck Back Hind quarter Tail base Chest	314 37 37 3 6 3	78.5 9.3 9.3 0.8 1.5 0.8
Skin scars	Absent Neck Back Hind quarter Tail base Chest	297 25 50 8 12 8	74.3 6.2 12.5 2.0 3.0 2.0
Coat condition	Clean Dirty	188 212	47.0 53.0
External parasites	Present Absent	202 198	50.5 49.5
Hoof health	Adequate	300	75.0
	Inadequate	100	25.0
Gait abnormality	Present	98	24.5
	Absent	302	75.7
Orifice discharges	Absent	229	57.3
	Nasal	81	20.2
	Mouth	18	4.5
	Ocular	72	18.0

**Table 5:** Summary of owner/user (n = 400) interview responses (indirect indicators).

Variable	Categories	Number	Percentage (%)
Sex	Male Female	383 17	95.8 4.2
Age	19–25 26–40 41–55 >55	8 121 183 88	2.0 30.3 45.7 22.0
Horse’s working days per week	1–2 days 3–4 days All days of the week	35 118 247	8.8 29.5 61.7
Horse work type	People transportation by cart Goods transportation by cart Riding Agricultural purposes Others	131 105 117 33 12	32.8 26.3 29.3 8.3 3.5
Daily feeding frequency	Once Twice More than twice	1 57 342	0.3 14.2 85.5
Daily watering frequency	Once Twice More than twice	19 198 183	4.8 49.5 45.7
Method/tool to encourage horse movement	Stick Whip Voice Hand	18 308 71 3	4.5 77.0 17.8 0.7
Trimming practice	Every 15 days	6	1.5
	Between 15 and 30 days	52	13.0
	>30 days	107	26.8
	Not done	235	58.7
Shoeing practice	Between 1 and 2 months	5	1.3
	>2 months	13	3.2
	Not done	382	95.5
Person responsible for shoeing	Farrier	11	2.8
	Owner	7	1.8
	None	382	95.4
Veterinary consultation	Never	178	44.5
	<1 year	150	37.5
	>1 year	72	18.0

**Table 6:** Summary findings on the behavioral status of the horses (n = 400).

Variable	Level	Number	Percentage (%)
General attitude	Alert Depressed	262 138	65.5 34.5
Approximation test	Indifference Friendliness Avoidance Aggressiveness	220 110 54 16	55.0 27.5 13.5 4.0
Walk-down-the-side	Indifference Friendliness Avoidance Aggressiveness	224 105 58 13	56.0 26.3 14.5 3.2
Chin contact	Accept Avoid	287 113	71.8 28.2
Biting	Yes No	20 380	5.0 95.0
Kicking	Yes No	36 364	9.0 91.0

## Data Availability

The original contributions presented in this study are included in the article. Further inquiries can be directed to the corresponding authors.
